# Covalent *N*-arylation by the pollutant 1,2-naphthoquinone activates the EGF receptor

**DOI:** 10.1016/j.jbc.2021.100524

**Published:** 2021-03-08

**Authors:** Kengo Nakahara, Kyohei Hamada, Tomoki Tsuchida, Nobumasa Takasugi, Yumi Abiko, Kazuhiko Shien, Shinichi Toyooka, Yoshito Kumagai, Takashi Uehara

**Affiliations:** 1 Department of Medicinal Pharmacology, Graduate School of Medicine, Dentistry and Pharmaceutical Sciences, Okayama University, Okayama, Japan; 2 Environmental Biology Laboratory, Faculty of Medicine, University of Tsukuba, Ibaraki, Japan; 3 Department of Thoracic, Breast and Endocrinological Surgery, Okayama University Graduate School of Medicine, Dentistry and Pharmaceutical Sciences, Okayama, Japan

**Keywords:** epidermal growth factor receptor, cell signaling, chemical modification, signal transduction, apoptosis, 1,2-NQ, 1,2-naphthoquinone, Akt, protein kinase B, BAD, B-cell lymphoma 2–associated death promoter, ErbB1, Erb-B1 receptor tyrosine kinase 1, ErbB2, Erb-B2 receptor tyrosine kinase 2, EGFR, epidermal growth factor receptor, hEGFR, human EGFR, HER, human epidermal growth factor receptor, IGF-1R, insulin-like growth factor 1 receptor, IR, insulin receptor, PARP, poly(ADP-ribose) polymerase, PDK1, phosphoinositide-dependent protein kinase-1, PM, particulate matter, PTP1B, protein-tyrosine phosphatase 1B, RTK, receptor tyrosine kinase, TSC2, tuberous sclerosis complex 2, UPLC–MSE, ultra–high-performance liquid chromatography–tandem mass spectrometry

## Abstract

The epidermal growth factor receptor (EGFR) is the most intensively investigated receptor tyrosine kinase. Several EGFR mutations and modifications have been shown to lead to abnormal self-activation, which plays a critical role in carcinogenesis. Environmental air pollutants, which are associated with cancer and respiratory diseases, can also activate EGFR. Specifically, the environmental electrophile 1,2-naphthoquinone (1,2-NQ), a component of diesel exhaust particles and particulate matter more generally, has previously been shown to impact EGFR signaling. However, the detailed mechanism of 1,2-NQ function is unknown. Here, we demonstrate that 1,2-NQ is a novel chemical activator of EGFR but not other EGFR family proteins. We found that 1,2-NQ forms a covalent bond, in a reaction referred to as *N*-arylation, with Lys80, which is in the ligand-binding domain. This modification activates the EGFR–Akt signaling pathway, which inhibits serum deprivation–induced cell death in a human lung adenocarcinoma cell line. Our study reveals a novel mode of EGFR pathway activation and suggests a link between abnormal EGFR activation and environmental pollutant–associated diseases such as cancer.

The epidermal growth factor receptor (EGFR), called Erb-B2 receptor tyrosine kinase 1 (ErbB1)/human epidermal growth factor receptor 1 (HER1), is the most well-established receptor tyrosine kinase (RTK), and overactivation of the EGFR signaling pathway is strongly linked to various types of cancer, such as non–small-cell lung cancer, breast cancer, and glioblastoma (1). EGFR activation initiates signal transduction through intracellular pathways, such as the extracellular signal–regulated kinase/mitogen-activated protein kinase, PI3K–protein kinase B (Akt), and Janus kinase/signal transducer and activator of transcription pathways, which result in oncogenic processes, such as cell proliferation, migration, and apoptosis resistance (2). In addition to genetic mutation of EGFR (3), genetic mutation–independent EGFR activation also contributes to cancer (4). Another group demonstrated that aberrant phosphorylation of EGFR by its ligands, such as epithelial cell adhesion molecule, is associated with cancer progression (5). Some studies have demonstrated that exposure to air pollutants is correlated with the incidence of lung and oral cancers (6). Furthermore, a recent study reported that polycyclic aromatic hydrocarbons, which are the major class of air pollutants, exhibit tumor-promoting effects (7). Although environmental factors such as particulate matter (PM) with a diameter of <2.5 μm (PM2.5) induce the phosphorylation of EGFR (8), the relationship between environmental factors and EGFR overactivation remains unclear.

Naphthalene containing gasoline is a major polycyclic aromatic hydrocarbon in the atmosphere (9), and its oxidation products such as naphthoquinones (NQs) have become a focus of interest because of their chemical properties based on forming covalent bonds and acting as electron transfer agents in redox reactions (10). Humans are exposed to NQs through combustion of fossil (11) and diesel fuel (12) and from tobacco smoke (13). NQs are reported to be formed from naphthalene through incomplete combustion and photooxidation of naphthalene in the atmosphere (14, 15) and the metabolic activation of naphthalene mediated by a variety of xenobiotic metabolizing enzymes in the body (Fig. S1) (10). In 2004, we first identified 1,2-naphthoquinone (1,2-NQ) as its diacetyl derivative from diesel exhaust particles and atmospheric PM2.5 and established a method for quantifying 1,2-NQ by gas chromatography–MS (16). Then, we also reported that levels of 1,2-NQ in the particulate phase and gas phase at different sites in Southern California were 5.8 to 246 pg/m^3^ and 109 to 445 pg/m^3^, respectively (10). Delgado-Saborit *et al.* (17) also found that the total (particulate and gas phases) concentration of 1,2-NQ was 3374 pg/m^3^ in a trafficked site in Birmingham, United Kingdom. These suggest that 1,2-NQ is an abundant ambient quinone derived from naphthalene, and atmospheric concentrations of 1,2-NQ are dependent on site collected and equipment used. Importantly, 1,2-NQ is particularly interesting because of two toxicologically relevant reactions. First, it acts as an electrophilic molecule and forms a covalent bond, a reaction referred to as *N*- or *S*-arylation, with target proteins. For example, endothelial nitric oxide synthase (18) and peroxiredoxin 6 (19) are known targets of 1,2-NQ; binding of 1,2-NQ to these proteins results in disruption of the redox state and the associated intracellular signaling pathway (20). In addition, 1,2-NQ can act as an electron donor to produce reactive oxygen species through redox cycling (10). Intriguingly, we previously demonstrated that 1,2-NQ phosphorylates EGFR by inactivating protein-tyrosine phosphatase 1B (PTP1B) (21), a negative regulator of EGFR (22). These lines of evidence indicate that 1,2-NQ, which is a component of the air pollutants, affects intracellular signal transduction, which may lead to carcinogenesis. However, the detailed mechanisms by which 1,2-NQ disrupts cellular homeostasis and promotes oncogenic effects remain unclear.

In the present study, we show that 1,2-NQ directly forms a covalent bond with Lys80, located in extracellular domain I of EGFR, and activates EGFR–Akt signaling. In addition, 1,2-NQ exerts a cytoprotective effect against serum deprivation–induced apoptotic cell death. Our study suggests a novel chemical modification of oncoproteins by air pollutants that contribute to tumorigenesis through dysregulation of intracellular signaling pathways.

## Results

### 1,2-NQ activates Akt *via* the PI3K/phosphoinositide-dependent protein kinase-1 pathway

**Figure 1:**
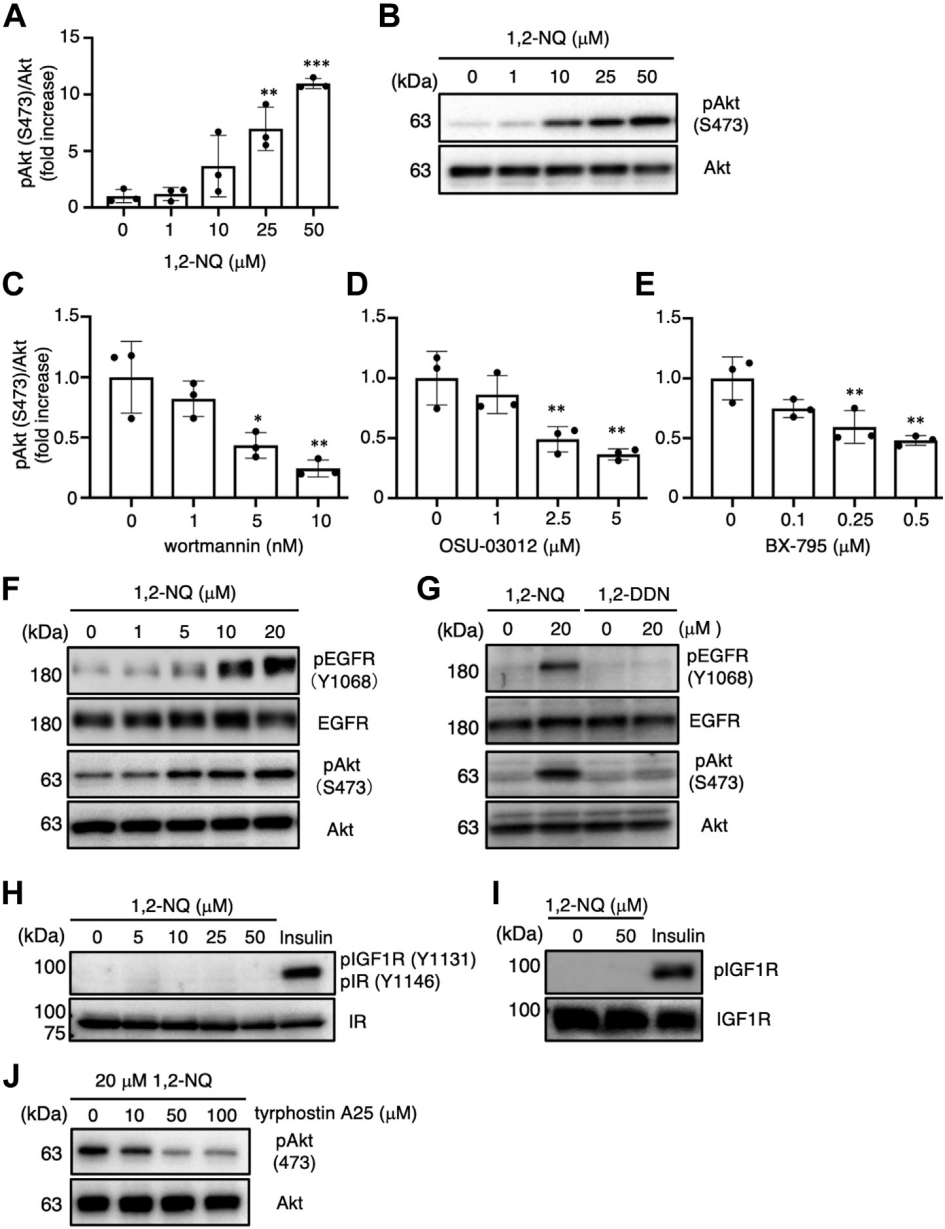
**Activation of Akt by 1,2-NQ *via* the PI3K–phosphoinositide-dependent protein kinase-1 pathway in A549 cells.***A* and *B*, cells were incubated with serum-free medium for 24 h at 37 °C and exposed to the indicated concentrations of 1,2-NQ for 15 min. The lysates were analyzed by Western blotting with anti-Akt or anti-phosphorylated Akt antibodies. *C*–*E*, cells were treated with the indicated concentrations of wortmannin (*C*), OSU-03012 (*D*), or BX-795 (*E*) for 10 min, 4 h, and 12 h, respectively, and were then stimulated with 20 μM 1,2-NQ for 15 min. *F*, cells were cultured with serum-free medium for 24 h prior to treatment with indicated concentrations of 1,2-NQ. *G*–*I*, cells were cultured with serum-free medium for 24 h prior to treatment with 20 μM 1,2-NQ, 1,2-DDN (*G*), or insulin (*H* and *I*) for 15 min. The levels of phosphorylated EGFR, total EGFR, phosphorylated IGF-1R, total IGF-1R, phosphorylated insulin receptor, and total insulin receptor were evaluated by Western blotting. *J*, cells were treated with the indicated concentrations of tyrphostin A25 for 12 h before stimulation with 20 μM 1,2-NQ. The cell lysates were then analyzed by Western blotting. Statistical analysis was carried out by one-way ANOVA with Bonferroni’s multiple comparisons test. All data are expressed as the mean ± SEM values. n = 3, ∗*p* < 0.05, ∗∗*p* < 0.01, and ∗∗∗*p* < 0.001 *versus* control. 1,2-DDN, 1,2-dihydronaphthalene; 1,2-NQ, 1,2-naphthoquinone; Akt, protein kinase B; EGFR, epidermal growth factor receptor; IGF-1R, insulin-like growth factor 1 receptor.

To explore the effect of 1,2-NQ on oncogenic cellular processes, we sought to determine whether 1,2-NQ affects the level of phosphorylated Akt, an active form of Akt, in human lung adenocarcinoma A549 cells. We analyzed the change in Akt phosphorylation in A549 cells. Akt was activated by 1,2-NQ treatment in a concentration-dependent manner (Fig. 1, *A* and *B*). Akt is phosphorylated by the PI3K/phosphoinositide-binding protein (phosphoinositide-dependent protein kinase-1 [PDK1]) pathway (23). To determine whether 1,2-NQ activates Akt *via* the PI3K/PDK1 pathway, we investigated the effect of specific inhibitors of PI3K and PDK1 on 1,2-NQ–induced Akt phosphorylation. Pretreatment with wortmannin (a PI3K inhibitor), OSU-03012 (a PDK1 inhibitor), or BX-795 (another PDK1 inhibitor) diminished the 1,2-NQ–mediated phosphorylation of Akt in a concentration-dependent manner (Fig. 1, *C*–*E* and Fig. S2, *A*–*C*). These data indicate that 1,2-NQ induces the phosphorylation of Akt *via* the PI3K/PDK1 pathway.

PI3K–Akt signaling is initiated by growth factors, such as EGF, insulin, and other extracellular stimuli (24). To determine the upstream target of 1,2-NQ in the PI3K/PDK1 pathway, we focused on the effect of 1,2-NQ on RTK family members, such as EGFR, the insulin receptor (IR), and the insulin-like growth factor 1 receptor (IGF-1R). To assess the activation of EGFR, we evaluated the phosphorylation level of EGFR after 1,2-NQ treatment. Exposure to 1,2-NQ enhanced the phosphorylation of EGFR in a concentration-dependent manner (Fig. 1*F* and Fig. S2*D*). As previously reported (21), *trans*-1,2-dihydroxy-1,2-dihydronaphthalene, a nonelectrophilic analog of 1,2-NQ, failed to activate EGFR/Akt signaling (Fig. 1*G* and Fig. S2, *E* and *F*). Therefore, the electrophilicity of 1,2-NQ plays an indispensable role in its ability to activate EGFR–Akt signaling. In addition, the phosphorylation of EGFR evoked by 1,2-NQ was observed in medium with or without serum (Fig. S2*G*). On the other hand, the phosphorylation of Akt was already detected in the media containing serum, and the enhancement of phosphorylated Akt formation by 1,2-NQ was only detected in the serum-free medium but not in serum-containing medium (Fig. S2*G*). Although serum contains some factors that can activate Akt, it might be irrelevant to the reactivity of 1,2-NQ to EGFR.

We previously showed that 1,2-NQ modifies and inactivates PTP1B, which acts as a negative regulator of RTKs, indicating that inactivation of PTP1B by 1,2-NQ partly contributes to the activation of EGFR (21). Surprisingly, although both IGF-1R and IR are also regulated by PTP1B (22), exposure to 1,2-NQ failed to induce the phosphorylation of these RTKs (Fig. 1, *H* and *I*). Consistent with this evidence, the selective EGFR kinase inhibitor tyrphostin A25 suppressed 1,2-NQ-induced phosphorylation of Akt in a concentration-dependent manner (Fig. 1*J* and Fig. S2*H*). These data indicate that 1,2-NQ may not only modify PTP1B but also directly activate EGFR.

**Figure 2:**
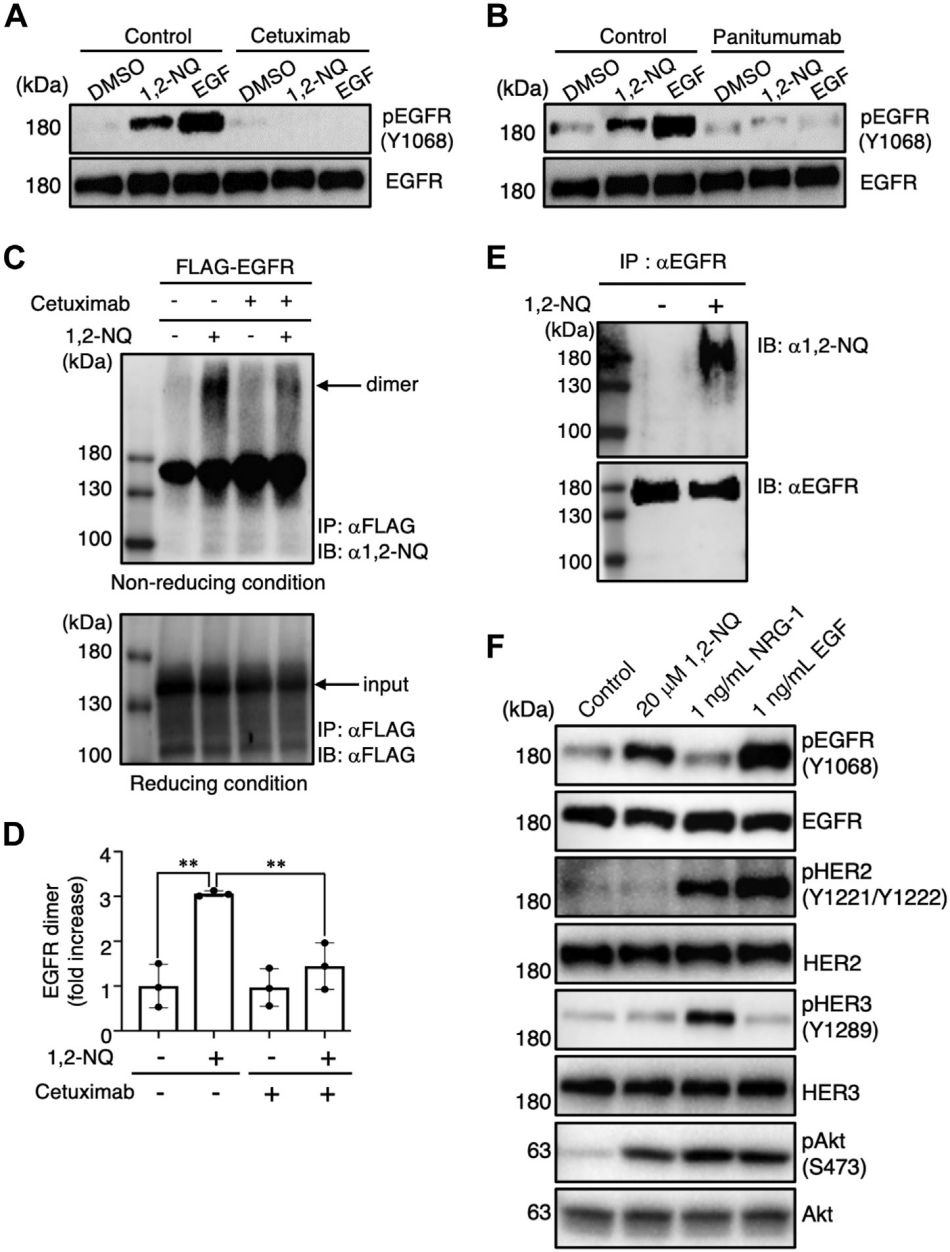
**1,2-NQ directly induces EGFR dimerization and tyrosine phosphorylation.***A* and *B*, A549 cells were incubated with serum-free medium for 24 h and were then exposed to 10 μM 1,2-NQ or 10 ng/ml EGF for 10 min. Cells were pretreated with cetuximab (*A*) or panitumumab (*B*) for 3 h before stimulation with 1,2-NQ or EGF. *C*, human embryonic kidney 293T cells were transiently transfected with pIDT-SMART (C-TSC) human EGFR-FLAG. Cells were incubated with serum-free medium for 18 h and were then exposed to 10 μM 1,2-NQ for 10 min. Cells were pretreated with cetuximab for 3 h before stimulation with 1,2-NQ. Immunoprecipitation was performed under nonreducing conditions for the detection of EGFR dimerization (*upper*) and under reducing conditions for input analysis (*lower*). Western blotting was conducted with anti-1,2-NQ or anti-FLAG antibodies. *D*, quantification of 1,2-NQ–induced EGFR dimerization in A549 cells. The relative level of EGFR dimers was normalized to the input EGFR level and shown as a fold change compared with the control. Statistical analysis was carried out by one-way ANOVA with Bonferroni's multiple comparisons test. All data are expressed as the mean ± SEM values. n = 3, ∗*p* < 0.05, and ∗∗*p* < 0.01 *versus* control. *E*, A549 cells were cultured with serum-free medium for 24 h before exposure to 10 μM 1,2-NQ for 10 min. Cell lysates were analyzed by immunoprecipitation using an anti-EGFR antibody and by Western blotting with anti-1,2-NQ or anti-EGFR antibodies. *F*, A549 cells were incubated with serum-free medium for 24 h and were then exposed to 20 μM 1,2-NQ, 1 ng/ml EGF, or 1 ng/ml NRG-1 for 10 min. 1,2-NQ, 1,2-naphthoquinone; Akt, protein kinase B; DMSO, dimethyl sulfoxide; EGF, epidermal growth factor; EGFR, epidermal growth factor receptor; HER, human epidermal growth factor receptor; NRG-1, neuregulin-1.

To further explore the regulatory mechanism of 1,2-NQ, we used the EGFR antagonist cetuximab, which can occupy the ligand-binding site in EGFR (25, 26). We found that 1,2-NQ–induced EGFR activation was dramatically diminished by pretreatment with either cetuximab (Fig. 2*A*) or panitumumab, which shares an epitope with cetuximab (Fig. 2*B*). Since EGFR dimerization is required for its tyrosine kinase activity (27), we investigated whether 1,2-NQ induces EGFR dimerization. Intriguingly, 1,2-NQ induced EGFR dimerization, which was decreased by pretreatment with cetuximab (Fig. 2, *C* and *D*). From these lines of evidence, we hypothesized that 1,2-NQ might directly bind and activate EGFR. To test this hypothesis, we conducted an immunoprecipitation assay using specific antibodies against EGFR and 1,2-NQ (28) and revealed that 1,2-NQ directly interacts with EGFR (Fig. 2*E*). On the other hand, it has been previously reported that EGF stimulation in cancer cells can promote both the homodimerization of EGFR and heterodimerization of EGFR with human epidermal growth factor receptor 2 (HER2)/Erb-B2 receptor tyrosine kinase 2 (ErbB2) (29). Alternatively, some reports indicated that EGF stimulates only homodimerization of EGFR, even though the phosphorylation of HER2 was evident (30, 31). In A549 cells, we detected the apparent and very weak formation of homodimerization of EGFR and the heterodimerization of EGFR/HER2 by treatment with EGF, respectively (Fig. S3). We then investigated the ability of 1,2-NQ in stimulating heterodimerization of EGFR with HER2/ErbB2. In A549 cells, we detected the expression of EGFR (HER1/ErbB1), HER2/ErbB2, and human epidermal growth factor receptor 3 (HER3)/ErbB3, but not ErbB4, by Western blotting (Fig. 2*F*). Then, we confirmed the effect of neureglin-1, a ligand for HERs but not EGFR, on the phosphorylation of each receptor. As shown in Figure 2*F*, treatment with neureglin-1 resulted in the significant phosphorylation of HER2 and HER3. Interestingly, challenge with 1,2-NQ specifically stimulated the phosphorylation of EGFR. In addition, 1,2-NQ could promote the homodimerization of EGFR but not heterodimerization of EGFR with HER2/ErbB2 (Fig. S3). Thus, these results indicate that 1,2-NQ can stimulate only the phosphorylation of EGFR and the formation of homodimer (EGFR) in A549 cells.

### 1,2-NQ forms *N*-aryl bonds with EGFR to induce EGFR self-activation

**Figure 3:**
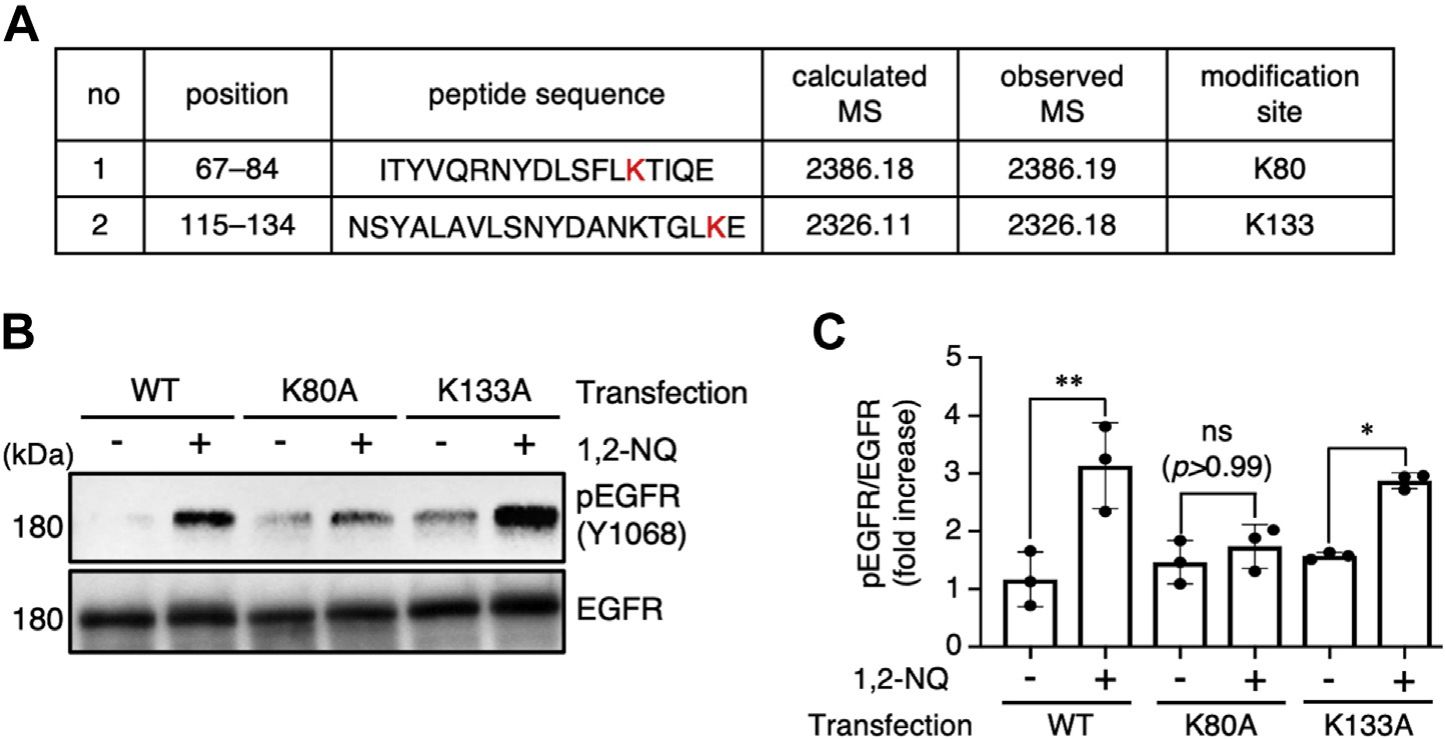
**1,2-NQ forms *N*-arylation with EGFR at Lys80 and induces its phosphorylation.***A*, identification of 1,2-NQ–binding sites in recombinant extracellular EGFR by ultra–high-performance liquid chromatography–tandem mass spectrometry analysis. Recombinant EGFR (5 μM) was incubated with 25 μM 1,2-NQ for 15 min at 25 °C in 50 mM ammonium bicarbonate. Trypsin-digested peptides were analyzed by ultra–high-performance liquid chromatography–tandem mass spectrometry. The mass data are shown in Fig. S4. *B* and *C*, human embryonic kidney 293T cells were transiently transfected with WT, K80A, or K133A human EGFR. Cells were incubated with serum-free medium for 18 h after 6 h of transfection and were then stimulated with 10 μM 1,2-NQ for 10 min. The relative level of phosphorylated EGFR was normalized to the level of total EGFR. Statistical analysis was carried out by one-way ANOVA with Bonferroni's multiple comparisons test. All data are expressed as the mean ± SEM values. n = 3, ∗*p* < 0.05, and ∗∗*p* < 0.01 *versus* control. 1,2-NQ, 1,2-naphthoquinone; EGFR, epidermal growth factor receptor; ns, not significant.

Protein nucleophiles, such as cysteine thiolates, histidine imidazoles, and lysine amines, are reactive to form *S*- or *N*-aryl bonds with electrophiles with α,β-unsaturated carbonyl groups, including 1,2-NQ. 1,2-NQ covalently modifies cysteine or histidine residues in its target proteins, for example, Kelch-like ECH-associated protein 1 (32) and IκB kinase β (33). From these lines of evidence, we speculated that 1,2-NQ forms either *S*- or *N*-aryl bonds with EGFR. To identify the 1,2-NQ modification sites in EGFR, we subjected the extracellular domain of 1,2-NQ–treated recombinant EGFR to trypsin digestion and then subjected the obtained peptides to ultra–high-performance liquid chromatography–tandem mass spectrometry (UPLC–MS^E^) analysis. UPLC–MS^E^ analysis revealed that the 1,2-NQ modified the lysine (Lys, K) 80 and Lys133 residues (Fig. 3*A* and Fig. S4). These lysine residues are located in EGFR extracellular domain I, which interacts with EGF (34). To determine whether these modification sites are essential for 1,2-NQ–induced EGFR phosphorylation, we substituted each lysine residue with an alanine (Ala, A). We found that 1,2-NQ failed to induce the phosphorylation of the EGFR K80A mutant but not the K133A mutant (Fig. 3, *B* and *C*). These results suggest that the modification of K80 but not K133 in EGFR is indispensable for EGFR–Akt pathway activation by 1,2-NQ. Also, we compared the amino acid sequence of the EGFR family members (Fig. S5). There are many lysine residues present in the extracellular domains of all EGFR family members. However, target lysine residues (K80 and 133) in EGFR do not exist in other types of EGFR members. Therefore, we concluded that 1,2-NQ interacts with EGFR (ErbB1) specifically through Lys80.

A previous study demonstrated that EGF phosphorylates EGFR for up to 180 min, whereas 1,2-NQ induces persistent activation of EGFR for up to 720 min (21). Based on a report stating that *N*-arylated 1,2-NQ is more resistant to reducing agents than *S*-arylated 1,2-NQ (35), we hypothesized that 1,2-NQ could form irreversible *N*-aryl bonds with EGFR and induce prolonged EGFR activation. To test this hypothesis, we evaluated the effect of GSH and DTT, another reducing agent, on EGFR modification by 1,2-NQ. Neither of these agents reversed the binding of 1,2-NQ to EGFR (Fig. S6). From these data, we speculated that *N*-arylation of EGFR by 1,2-NQ might contribute to the persistent disruption in its protein function.

**Figure 4:**
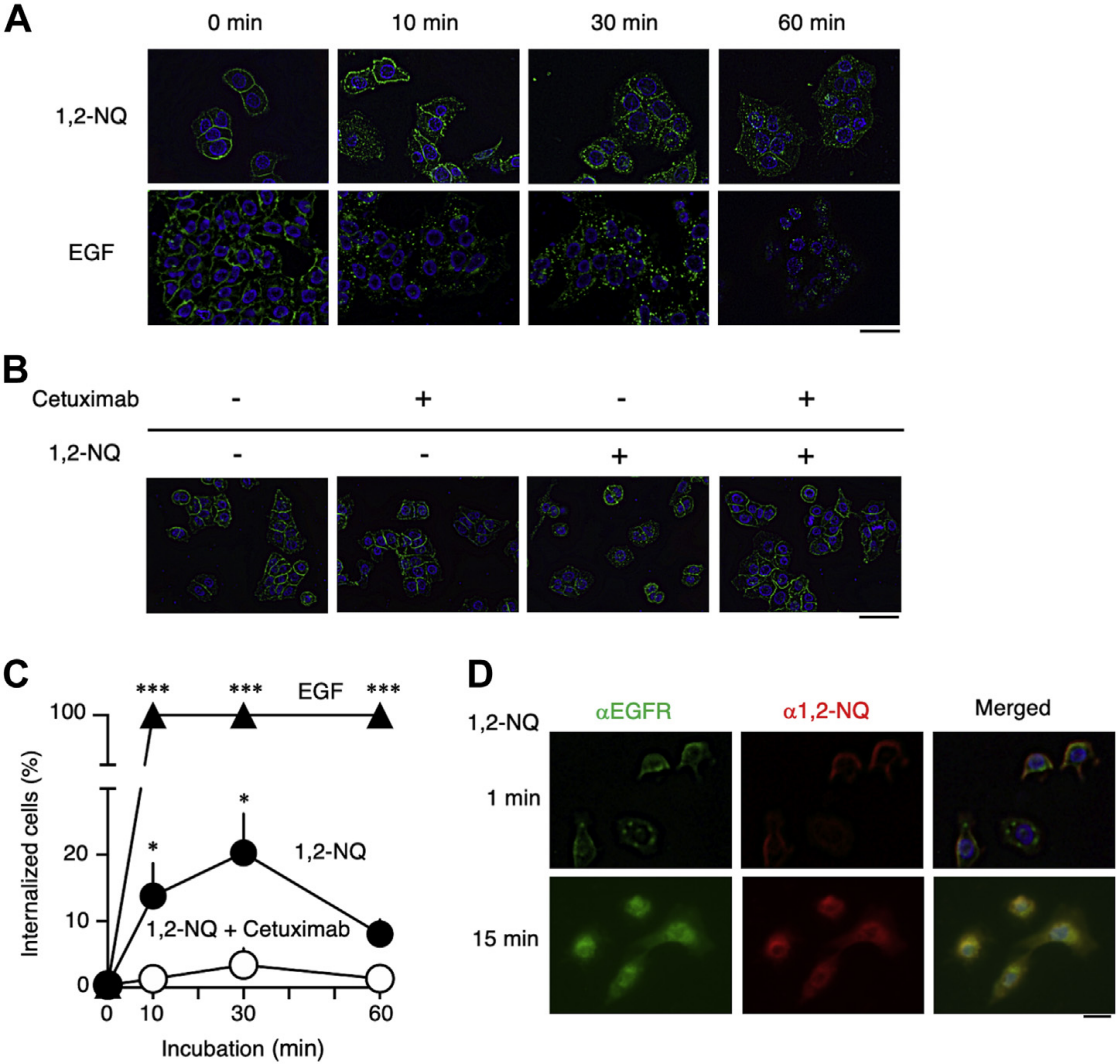
**1,2-NQ induces EGFR internalization.***A*, cells were incubated with serum-free medium for 3 h and were then stimulated with 10 μM 1,2-NQ for the indicated time. Immunofluorescence staining was performed to visualize EGFR internalization. The scale bar represents 50 μm. *B*, cells were pretreated with cetuximab in serum-free medium for 3 h and were then exposed to 1,2-NQ for 10 min. Immunofluorescence staining was performed. The scale bar represents 50 μm. *C*, quantification of EGFR internalization by exposure to 1,2-NQ or EGF for the indicated time. A549 cells were cultured in serum-free medium for 3 h and were then exposed to 1,2-NQ or EGF. 1,2-NQ-treated cells were pretreated with cetuximab in serum-free medium for 3 h. Statistical analysis was carried out by two-way ANOVA with Bonferroni’s multiple comparisons test. The data are expressed as the mean ± SEM values. n = 3, ∗*p* < 0.05 *versus* control. *D*, cells were cultured in serum-free medium for 3 h and were then stimulated with 10 μM 1,2-NQ for the indicated time. Immunofluorescence staining was conducted to visualize the colocalization of EGFR and 1,2-NQ. 1,2-NQ, 1,2-naphthoquinone; EGF, epidermal growth factor; EGFR, epidermal growth factor receptor.

EGFR signaling is strictly regulated by its internalization and subsequent localization in endosomes, leading to its recycling or degradation (36). Hence, we examined the effect of 1,2-NQ on EGFR internalization. While EGFR localized at the plasma membrane under basal conditions, treatment with its natural ligand EGF induced the formation of EGFR puncta, indicating EGFR internalization (Fig. 4*A*, lower panel). Interestingly, 1,2-NQ also induced the formation of EGFR puncta, similar to EGF treatment (Fig. 4*A*, upper panel). In addition, 1,2-NQ–induced EGFR internalization was also suppressed by pretreatment with cetuximab (Fig. 4, *B* and *C*). Furthermore, we confirmed by an immunofluorescence assay that 1,2-NQ colocalizes with EGFR (Fig. 4*D*). These data support the hypothesis that 1,2-NQ interacts with EGFR and induces its internalization *via N*-arylation.

### 1,2-NQ exerts antiapoptotic effects *via* EGFR–Akt signaling

**Figure 5:**
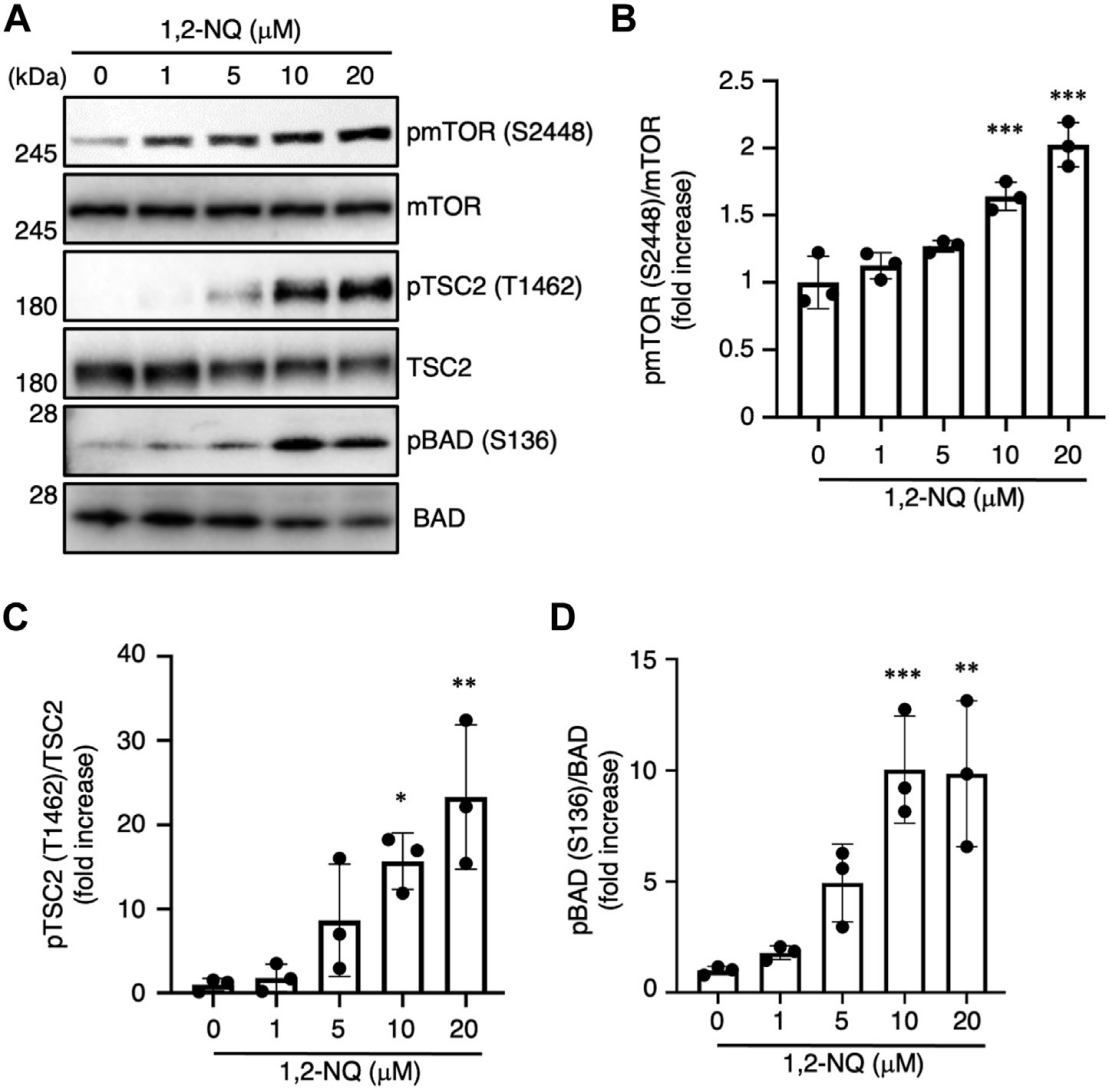
**1,2-NQ activates the downstream of Akt signaling pathway in A549 cells.***A*–*D*, cells were incubated with serum-free medium for 24 h before exposure to 1,2-NQ for 30 min. The lysates were analyzed by Western blotting with anti-mTOR, anti-phosphorylated mTOR, anti-TSC2, anti-phosphorylated TSC2, anti-BAD or anti-phosphorylated BAD antibody. Statistical analysis was carried out by one-way ANOVA with Bonferroni's multiple comparisons test. The data are expressed as the mean ± SEM values. n = 3, ∗*p* < 0.05, ∗∗*p* < 0.01, and ∗∗∗*p* < 0.001 *versus* control. 1,2-NQ, 1,2-naphthoquinone; BAD, B-cell lymphoma 2–associated death promoter; mTOR, mechanistic target of rapamycin; TSC2, tuberous sclerosis complex 2.

**Figure 6:**
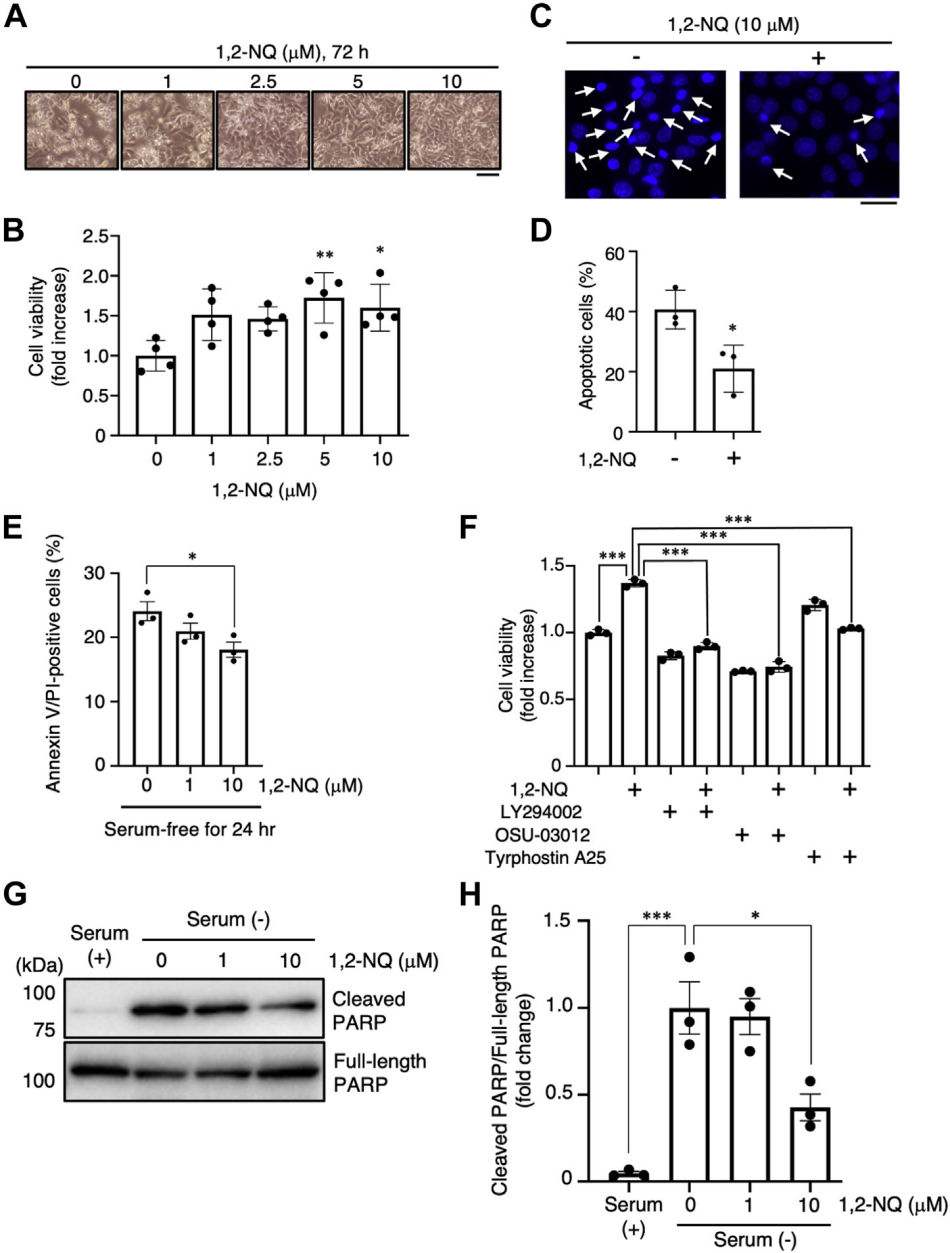
**The antiapoptotic effect of 1,2-NQ is mediated *via* the epidermal growth factor receptor–PI3K–Akt pathway in A549 cells.***A*–*D*, cells were stimulated with the indicated concentrations of 1,2-NQ for 10 min and were then incubated with serum-free medium. After 72 h, cells were observed using a phase contrast microscope (*A*), and cell viability was then determined by a water soluble tetrazolium salt assay (*B*). The scale bar represents 100 μm. Cells were treated with Hoechst 33342 for 30 min and were then observed using a fluorescence microscope. The *arrows* show the apoptotic cells. *C* and *D*, the scale bar represents 50 μm. The data are expressed as the mean ± SEM values. ∗*p* < 0.05 and ∗∗*p* < 0.01 *versus* control. *E*, cells were incubated with serum-free medium for 12 h and were then stimulated with 1,2-NQ at indicated concentrations for 10 min. After the incubation of serum-free medium for 24 h, cells were stained by annexin V–FITC/propidium iodide and then observed using a fluorescence microscope. Annexin V/propidium iodide double positive cells were counted as apoptotic cells. The data are expressed as the mean ± SEM values. ∗*p* < 0.05 *versus* 0 μM 1,2-NQ. *F*, cells were cultured with serum-free medium for 24 h and were then exposed to 1,2-NQ (10 μM) for 10 min after treatment with LY294002 (10 μM) and OSU-03012 (5 μM) for 12 h. Cells were treated with tyrphostin A25 (100 μM) in serum-free medium for 24 h after stimulation with 10 μM 1,2-NQ. After 72 h, cell viability was determined by a water soluble tetrazolium salt assay. *G* and *H*, cells were stimulated with 1,2-NQ for 10 min and were then incubated with serum-free medium for 24 h. The lysates were analyzed by Western blotting with anti-PARP or anticleaved PARP antibodies. Statistical analysis was carried out by two-way ANOVA with Bonferroni's multiple comparisons test. The data are expressed as the mean ± SEM values. ∗*p* < 0.05 and ∗∗∗*p* < 0.001 between the two indicated groups. 1,2-NQ, 1,2-naphthoquinone; PARP, poly(ADP-ribose) polymerase.

Activation of the EGFR–PI3K–Akt signaling pathway influences apoptosis resistance, glucose metabolism, and autophagy (2). Activated Akt can phosphorylate various substrates, such as tuberous sclerosis complex 2 (TSC2), mechanistic target of rapamycin (mTOR), and B-cell lymphoma 2–associated death promoter (BAD) (37, 38). It is known that these targets exert protein synthesis, cell growth, and an antiapoptotic effect. Therefore, we sought to determine whether 1,2-NQ can phosphorylate mTOR, TSC2, and BAD in A549 cells. 1,2-NQ induced the phosphorylation of mTOR, TSC2, and BAD in a concentration-dependent manner (Fig. 5, *A*–*D*). Other groups have reported that serum deprivation causes apoptotic death of A549 cells (39), which is inhibited by Akt activity (40). To assess the effect of 1,2-NQ on apoptotic stimuli, we incubated A549 cells with 1,2-NQ and induced apoptosis by treatment with serum-free medium, which deprives cells of growth factors, in order to suppress EGFR-dependent antiapoptotic effects (41). As a result, 1,2-NQ suppressed serum deprivation–induced cell death (Fig. 6, *A* and *B*). Apoptotic cells exhibiting nuclear condensation, a typical signature of apoptosis, were assessed by Hoechst staining and annexin V/propidium iodide (PI) staining, and we also confirmed the cytoprotective effect of 1,2-NQ (Fig. 6, *C*–*E*). In addition, we used specific inhibitors to examine whether 1,2-NQ exerts its antiapoptotic effect *via* the EGFR–Akt signaling pathway. Treatment with these inhibitors significantly abrogated the effect of 1,2-NQ (Fig. 6*F*). Finally, we evaluated that the effect of 1,2-NQ on the cleavage of poly(ADP-ribose) polymerase (PARP), by serum deprivation. As shown Figure 6, *G* and *H*, 1,2-NQ significantly inhibited the serum starvation–induced cleavage of PARP. These results strongly suggested that 1,2-NQ attenuated serum deprivation–induced apoptotic cell death through activation of EGFR–Akt pathway. Our data indicate that 1,2-NQ induces activation of the EGFR–Akt signaling pathway *via* binding directly to EGFR and that this modification renders cancer cells resistant to apoptotic stimuli.

## Discussion

To date, many studies have demonstrated that ambient pollutants can act as mutagens through the formation of DNA adducts (42), leading to carcinogenesis. On the other hand, few studies have indicated that chemical modification of proteins by air pollutants may lead to carcinogenesis. Therefore, understanding the effects of environmental factors on the regulation of oncogenic protein activity is important to prevent carcinogenesis. In the present study, we showed that a component of environmental air pollutants, 1,2-NQ, can activate EGFR *via N*-arylation at Lys80. We also revealed that 1,2-NQ treatment and aberrant activation of EGFR–Akt signaling promote apoptosis resistance in a cancer cell line, which may correlate with carcinogenesis.

Carcinogenesis originates from both genetic and environmental factors, indicating that these factors are related to carcinogenesis (43). Regarding genetic factors, many studies have demonstrated that gene mutations in EGFR are strongly linked to lung cancer through the disruption of intracellular signaling pathways (3). In addition, post-translational modifications of EGFR play an important role in regulating its activation, and dysregulation of these events may be associated with carcinogenesis. For example, peroxide-dependent sulfenylation of EGFR at Cys797 increases its kinase activity (44). Although we previously reported that 1,2-NQ modifies PTP1B at Cys121, leading to EGFR activation (21), here, we found that 1,2-NQ failed to activate the other RTKs, namely, IR and IGF-1R (Fig. 1, *H* and *I*). These data indicate that 1,2-NQ primarily induces phosphorylation in the EGFR–Akt signaling pathway *via N*-arylation of EGFR and that the modification of PTP1B by 1,2-NQ may synergistically contribute to the resulting signal transduction. UPLC–MS^E^ analysis revealed that 1,2-NQ binds to EGFR at Lys80 and Lys133 (Fig. 3*A*). In addition, the electrophilicity of 1,2-NQ is required for EGFR activation (Fig. 1*G*), indicating that 1,2-NQ forms a covalent bond *via* a Michael addition reaction. Protein nucleophiles such as cysteine thiolates can react with 1,2-NQ, leading to *S*-arylation, which is a reversible modification (45). On the other hand, previous report has shown that EGF stimulation in cancer cells promoted both the homodimerization of EGFR and heterodimerization of EGFR with HER2/ErbB2 (29). We then investigated whether 1,2-NQ stimulates the formation of homodimers/heterodimers. Interestingly, treatment with 1,2-NQ promoted the homodimerization of EGFR but not heterodimerization of EGFR with HER2/ErbB2. These results strongly suggested that 1,2-NQ induces the receptor formation of homodimers (EGFR) in A549 cells. Also, we compared the amino acid sequence of the EGFR family members. There are many lysine residues present in the extracellular domains of all EGFR family members. However, as shown in Fig. S5, target lysine residues (K80 and 133) in EGFR do not exist in other types of EGFR members. Therefore, we concluded that 1,2-NQ interacts with EGFR (ErbB1/HER1) specifically through Lys80.

1,2-NQ is expected to form adducts not only on EGFR but also other proteins. It is speculated that the adducts might reflect targets that are independent of their half-lives and be depend on the affinity of proteins to 1,2-NQ or the affinity of 1,2-NQ to proteins. On the other hand, lysine amines and histidine imidazoles can undergo *N*-arylation, which is an irreversible modification (35). Consistent with these findings, the covalent bond between 1,2-NQ and EGFR was resistant to reducing agents (Fig. S6). From these data, we speculated that *N*-arylation by 1,2-NQ might contribute to the persistent disruption in EGFR protein function. Consistent with this hypothesis, a previous study demonstrated that compared with EGF, 1,2-NQ prolongs EGFR activation (21). Since 1,2-NQ can activate the extracellular signal–regulated kinase signaling pathway *via* EGFR activation (46), we concluded that 1,2-NQ affects other intracellular signaling pathways, resulting in disruption of cellular homeostasis.

EGFR comprises four extracellular domains and an intracellular region containing the tyrosine kinase domain. Its ligands, for example, EGF, bind with extracellular domains I and III, leading to dimerization and autophosphorylation of EGFR (27). UPLC–MS^E^ and biochemical analyses revealed that 1,2-NQ binds with EGFR at Lys80 (Fig. 3*A*), indicating that Lys80 in EGFR is indispensable for 1,2-NQ–induced EGFR activation. Interestingly, the endogenous ligand EGF binds with EGFR *via* domains I and III, whereas 1,2-NQ binds only with domain I and can induce the phosphorylation of EGFR. We confirmed that no mutations or variants at Lys80, which is associated with cancer, are reported in the Catalogue Of Somatic Mutations In Cancer (https://cancer.sanger.ac.uk/cosmic) (47). However, another study reported that mutations in extracellular domains I (R108K) and II (A209V) of EGFR in glioblastoma lead to ligand-independent phosphorylation (48). These mutations may lead to conformational changes in the extracellular regions of EGFR, resulting in the induction of ligand-independent EGFR phosphorylation (48, 49). Furthermore, the G33K and N56K mutations, which are also located in domain I, cause ligand-independent EGFR phosphorylation in head and neck cancer (50). These mutations may also cause abnormal phosphorylation by altering EGFR structural dynamics. These reports suggest that conformational changes in the extracellular regions of EGFR can affect its phosphorylation and signal transduction. Although further structural studies are needed to understand the exact mechanisms, we speculate that *N*-arylation of K80 by 1,2-NQ causes a conformational change in EGFR and then induces ligand-independent EGFR phosphorylation.

We also elucidated whether 1,2-NQ exerted an antiapoptotic effect in A549 cells to protect against serum deprivation (Fig. 6). In the early stage of carcinogenesis, cancer cells are exposed to nutrient deprivation because of insufficient vascularization (51). Our results showed that the number of annexin V/PI-positive cells, which indicates apoptosis, significantly decreased after treatment with 1,2-NQ. This result together with the data on cell viability, morphological changes, and Hoechst staining suggested that 1,2-NQ has the ability to inhibit cell death induced by serum starvation *via* EGFR–Akt signaling. Therefore, our data suggest that 1,2-NQ can promote the survival of cancer cells under nutrient-poor conditions by activating Akt signaling. These lines of evidence suggest that cigarette smoking may exacerbate respiratory diseases and carcinogenesis *via N*-arylation of EGFR.

It has been widely recognized that not only genetic mutation but also aberrant functional modification of EGFR contributes to the pathogenesis of cancer. However, the effect of environmental factors such as ambient air pollutants on EGFR activity remains poorly understood so far. In this study, we demonstrated that the air pollutant 1,2-NQ activates the EGFR–Akt signaling pathway (Fig. 1). In addition to our study, another recent study showed that exposure to PM2.5 containing 1,2-NQ increased the phosphorylation of EGFR and Akt in mice (52). Taken together, these findings indicate that the environmental air pollutant 1,2-NQ can induce EGFR–Akt signaling *via N*-arylation of EGFR, leading to the inhibition of serum starvation–induced apoptotic cell death. These data emphasize that *N*-arylation by 1,2-NQ is a novel aberrant chemical modification of EGFR and may contribute to carcinogenesis by inducing aberrant activation of EGFR–Akt signaling. To our knowledge, this evidence is the first to indicate that extracellular post-translational modifications regulate EGFR activity. Our study sheds light on the mechanism of EGFR overactivation by 1,2-NQ in environmental factor–associated diseases such as cancer.

## Experimental procedures

### Reagents and antibodies

Antibodies against Akt, phospho-Akt (S473) (D9E), EGFR (D38B1), phospho-EGFR (Y1068) (D7A5), HER2 (D8F12), phosphor-HER2 (Y1221/1222) (6B12), HER3 (D22C5), phosphor-HER3 (Y1289) (21D3), IR (4B8), phospho-IR (Y1146), IGF-IR (D23H3), p-IGF-IR (Y1131) (DA7A8), mTOR (7C10), phosphor-mTOR (S2448) (D9C2), TSC2 (D93F12), phosphor-TSC2 (T1462) (5B12), BAD (D24A9) and phospho-BAD (S136) (D25H8), PARP, and cleaved PARP (Asp214) (D64E10) were purchased from Cell Signaling Technology. Antibodies against FLAG (DYKDDDDK) were purchased from Sigma–Aldrich. Antibodies against 1,2-NQ were generated as previously described (21). OSU-03012 and LY294002 were purchased from ChemScene. Tyrphostin A25 was purchased from Wako. Recombinant human neuregulin-1 was purchased from Cell Signaling Technology. The expression vector for FLAG-tagged WT EGFR (pIDT-SMART [C-TSC] EGFR-FLAG) was prepared as previously described (53).

### Western blotting

Cells were washed with PBS and lysed in ice-cold radioimmunoprecipitation assay buffer (40 mM Tris–HCl [pH 8.0], 150 mM NaCl, 0.5% [w/v] sodium deoxycholate, 0.1% [w/v] SDS, 1% [v/v] Nonidet P-40, and a protease inhibitor cocktail) on ice for 5 min. After quantification *via* the bicinchoninic acid assay method, protein samples were boiled in 1× Laemmli SDS sample buffer (62.5 mM Tris–HCl [pH 6.8], 5% v/v 2-mercaptoethanol, 2% [w/v] SDS, and 10% [v/v] glycerol) for 5 min. Samples were subjected to Western blotting as previously described (54). For immunoprecipitation studies, cell lysates were incubated first with an anti-FLAG (M2) mAb (1:250) for 2 h at 4 °C and then with protein G Sepharose beads for 2 h at 4 °C. After wash with radioimmunoprecipitation assay buffer, samples were eluted from the beads with nonreducing or reducing 1× Laemmli SDS sample buffer.

### Cell culture and transfection

A549 human lung adenocarcinoma epithelial cells and human embryonic kidney 293T cells were cultured in Dulbecco's modified Eagle's medium supplemented with 10% (v/v) heat-inactivated fetal bovine serum at 37 °C in a humidified atmosphere of CO_2_/95% air. Transfections were conducted using polyethylenimine MAX (Polysciences, Inc) according to the manufacturer's instructions.

### UPLC–MS^E^

Recombinant human EGFR (hEGFR; Sino Biological, Inc; 5 μM) was incubated with dimethyl sulfoxide as a control or 25 μM 1,2-NQ for 15 min at 25 °C in 50 mM ammonium bicarbonate. A solution of Tris(2-carboxyethyl)phosphine (2.5 mM) in ammonium bicarbonate was added to the mixture and incubated for 20 min at 25 °C. Proteins in the mixture were alkylated by treatment with 5 mM 2-iodoacetamide at 25 °C in the dark for 20 min. hEGFR (3.45 μg) was digested with Glu-C (100 ng) for 16 h at 37 °C, and an aliquot of 1% (v/v) formic acid was then added to the resulting peptides. Analyses were performed using a nanoAcquity UPLC system (Waters Co) equipped with a BEH130 nanoAcquity C18 column (100 mm × 75 μm i.d., 1.7 μm) held at 35 °C according to procedures described previously, with slight modification (55). In brief, mobile phases A (water containing 0.1% [v/v] formic acid) and B (acetonitrile containing 0.1% [v/v] formic acid) were linearly mixed by using a gradient program. The flow rate was 0.3 μl/min, and the mobile phase composition was as follows: 3% B for 1 min, a linear increase over 74 min to 40% B, 40% B for 4 min, a linearly increase over 1 min to 95% B, and 95% B for 5 min. The eluted peptides were then transferred to the nanoelectrospray source of a quadrupole time-of-flight mass spectrometer (SYNAPT High Definition Mass Spectrometry System, Waters Co). The system was controlled, and mass spectra were analyzed with Waters MassLynx software, version 4.1 (Waters Co). An electrospray ionization source was used, with a capillary voltage of 2.5 kV and a sampling cone voltage of 35 V. A low (6 eV) or increasing (step from 30 to 45 eV) collision energy was used to generate either intact peptide precursor ions (low energy) or peptide productions (increasing energy). The source temperature was 100 °C, and the detector was operated in positive-ion mode. Data were collected in centroid mode from *m*/*z* 300 to 2000. All analyses were performed with an independent reference, Glu-1-fibrinopeptide B (*m*/*z* 785.8426). BiopharmaLynx, version 1.2 software (Waters) was used for baseline subtraction and smoothing, deisotoping, *de novo* peptide sequence identification, and database search against the sequence of hEGFR (NP_005219.2). The following default search settings were applied: enzyme, Glu-C; maximum missed cleavage, 3; variable modifications, carbamidomethyl (C); and 1,2-NQ (C, H, K); mass tolerance, ± 30 ppm; MS ion intensity threshold, 250 counts; MS^E^ ion intensity threshold, 100 counts. All peptides were filtered by applying a minimum intensity threshold of 5% to exclude peptides at low intensity. The matched masses of hEGFR with or without 1,2-NQ covered 100% of the hEGFR sequence. Peptide fragments containing 1,2-NQ were identified on the basis of their calculated *m/z* values (fragment +156.02). Their equivalent mass was added to the mass of the unmodified peptide as 1,2-NQ covalently binds protein thiol through the 1,4-Michael addition reaction to form 1,2-NQH_2_, which easily undergoes auto-oxidation to form protein-1,2-NQ.

### PCR-based site-directed mutagenesis

PCR-based site-directed mutagenesis was performed with PrimeSTAR Max DNA polymerase (Takara Bio Inc) using the following primer pairs: K80A EGFR forward, 5’-TTC TTA GCG ACC ATC CAG GAG GTG GC-3’ and K80A EGFR reverse, 5’-GAT GGT CGC TAA GAA GGA AAG ATC AT-3’; K133A EGFR forward, 5’-CCG GAC TGA GCG AGC TGC CCA TGA GAA ATT TA-3’ and K133A EGFR reverse, 5’-TGG GCA GCT CCG CCA GTC CGG TTT TAT TTG-3’. The PCR products were incubated with DpnI for 1 h at 37 °C. The mutated plasmid DNA was transformed into competent *Escherichia coli* DH5α cells. The mutated sequences were confirmed by Sanger sequencing.

### Immunocytochemistry

A549 cells were cultured on coverslips. After exposure to 1,2-NQ, cells were washed 3 times with PBS, fixed with 4% (v/v) paraformaldehyde in PBS for 20 min at RT, washed 3 times with PBS, and permeabilized with PBS-Triton-X (0.1% [v/v]) for 10 min at 4 °C. Cells were then blocked with 1% (w/v) bovine serum albumin in PBS for 1 h at RT and incubated with primary anti-EGFR and/or anti-1,2-NQ antibodies for 2 h at RT. Cells were washed 3 times with PBS and were then incubated with Alexa 488- or 594-conjugated goat anti-rabbit or mouse IgG antibodies (Invitrogen; 2 mg/ml, 1:400) for 30 min at RT. Coverslips were mounted with Vectashield with 4′,6-diamidino-2-phenylindole (Vector Laboratories, Inc) for 15 min at RT, fluorescence was examined using a fluorescence microscope (FSX100; Olympus). Cells containing puncta were counted as EGFR-internalized cells.

### Assessment of cell viability

Cell viability was measured in triplicate wells of 12-well plates by a Cell Counting Kit-8 (Dojindo). A549 cells were serum starved for 24 h before exposure to 1,2-NQ. Cells were treated with LY294002, OSU-03012, or tyrphostin A25 for 12 h or 24 h before stimulation with 1,2-NQ. After 72 h, water soluble tetrazolium salt reagents were added to cells in a 12-well plate, and the cells were incubated at 37 °C for 1 h. Cell viability was assessed by measuring the OD450. The number of apoptotic nuclei in A549 cells was determined by Hoechst 33342 staining and evaluation with a fluorescence microscope (FSX100; Olympus). Condensed nuclei were considered apoptotic nuclei.

### Annexin V/PI staining

Annexin V/PI staining was performed by the provided protocol of annexin V-FITC Apoptosis Detection Kit (Nacalai Tesque). Briefly, cells were incubated with serum-free medium for 24 h. 1,2-NQ was treated at the 12 h of serum-free incubation for 10 min and then washed out. Cells were incubated with the solution containing annexin V–FITC/PI at room temperature for 15 min. The samples were immediately observed by fluorescence microscope (BZ-X810; Keyence).

### Statistical analysis

All experiments were independently performed at least 3 times. All data are expressed as the mean ± SEM values. Statistical comparisons were conducted using ANOVA with post hoc Bonferroni correction or Student's *t* test in GraphPad Prism 7 (GraphPad Software). *p* Values of <0.05 were considered to indicate significant differences.

## Data availability

All data are contained within the article and the supporting information.

## Supporting information

This article contains supporting information.

## Conflict of interest

The authors declare that they have no conflicts of interest with the contents of this article.
