# Supplementary material for: Covalent N-arylation by the pollutant 1,2-naphthoquinone activates the EGF receptor
Source: J Biol Chem. 2021 Mar 8;296:100524. doi: 10.1016/j.jbc.2021.100524 (PMC8050034; doi:10.1016/j.jbc.2021.100524)
Supplement: Supplemental Figures S1–S6 [file mmc1.docx]

**Covalent *N*-arylation by the pollutant 1,2-naphthoquinone activates the EGF receptor**

Kengo Nakahara^1, †^, Kyohei Hamada^1, †^, Tomoki Tsuchida^1^, Nobumasa Takasugi^1^, Yumi Abiko^3^, Kazuhiko Shien^2^, Shinichi Toyooka^2^, Yoshito Kumagai^3^, & Takashi Uehara^1, *^

^1^*Department of Medicinal Pharmacology, Graduate School of Medicine, Dentistry and Pharmaceutical Sciences, Okayama University, Okayama 700-8530, Japan*

*^2^Department of Thoracic, Breast and Endocrinological Surgery, Okayama University Graduate School of Medicine, Dentistry and Pharmaceutical Sciences, Okayama, 700-8558, Japan*

^3^*Environmental Biology Laboratory, Faculty of Medicine, University of Tsukuba, Ibaraki 306-8575, Japan*

*To whom correspondence should be addressed: Takashi Uehara, Ph.D., email: [uehara-t@okayama-u.ac.jp](mailto:uehara-t@okayama-u.ac.jp);

^†^These authors contributed equally to this work.

**File Name:** Supporting Information

**Description:** 6 Supporting Figures


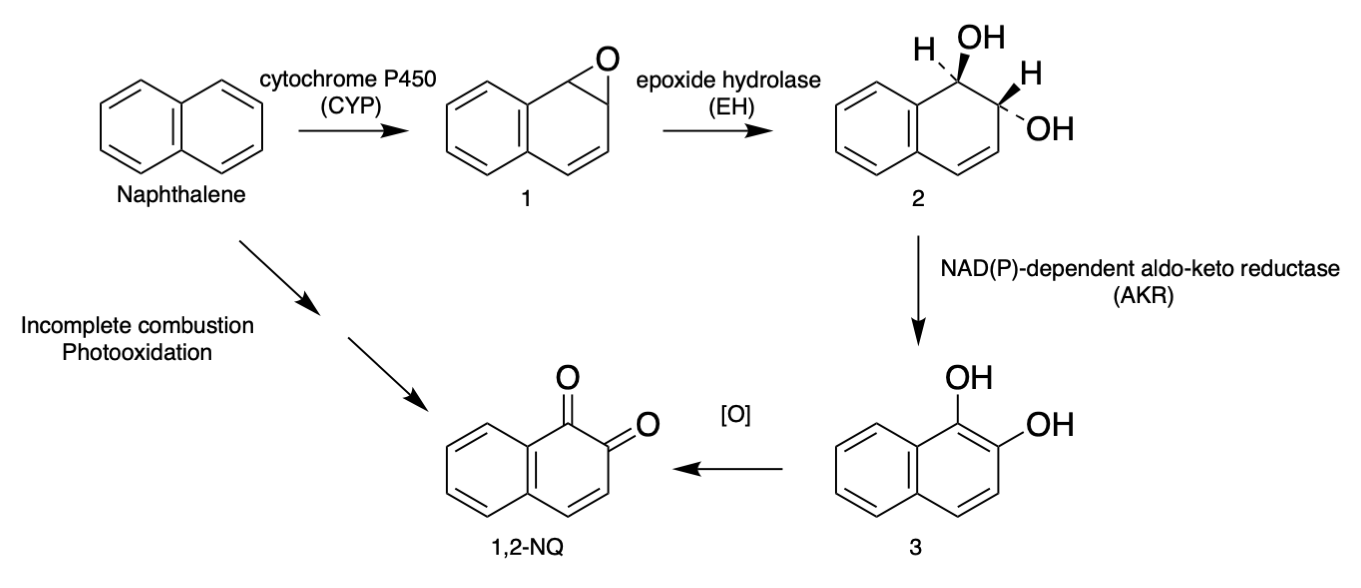


**Figure S1. Chemical-　and bio-transformations of 1,2-naphthoquinone (1,2-NQ) from naphthalene in the atmosphere and in the body.**

Metabolite 1, naphthalene 1,2-epoxide; metabolite 2, *trans*-1,2-dihydroxy-1,2-dihydro naphthalene; metabolite 3, 1,2-dihydroxynaphthalene.

**Figure S2. Activation of Akt by 1,2-NQ via the PI3K-PDK1 pathway.**

(*A-C*) Cells were treated with the indicated concentrations of wortmannin (*A*), OSU-03012 (*B*) or BX-795 (*C*) for 10 min, 4 h, and 12 h, respectively, and were then stimulated with 20 μM 1,2-NQ for 15 min. (*D*) Cells were treated with the indicated concentrations of 1,2-NQ for 15 min. The relative level of phosphorylated EGFR was normalized to the level of total EGFR. Statistical analysis was carried out by one-way ANOVA with Bonferroni’s multiple comparisons test. All data are expressed as the mean ± S.E.M. values. n=4, **p*<0.05, ****p*<0.001 vs. control. (*E, F*) Quantification of the effect of the electrophilicity of 1,2-NQ on 1,2-NQ-induced EGFR and Akt phosphorylation in A549 cells. All data are expressed as the mean ± S.E.M. values. n=3, ****p*<0.001 vs. control. (*G*) The effect of serum in the culture medium on activation of EGF signaling induced by 1,2-NQ. Cells were cultured with or without serum for 24 h, and then exposed to 1,2-NQ for 10 min. (*H*) Quantification of the effect of tyrphostin A25 on 1,2-NQ-induced Akt phosphorylation in A549 cells.

**Figure S3. Dimer formation of EGFR by 1,2-NQ.**

A549 cells were transiently transfected with pIDT-SMART (C-TSC) WT EGFR-FLAG and pcDNA6 WT EGFR-myc-His (1:1). After 6 h of transfection, cells were incubated with serum-free medium for 24 h and were then exposed to 20 μM 1,2-NQ or 1 ng/mL EGF at room temperature for 1 min. Cell lysates were subjected to immunoprecipitation assay using anti-FLAG, anti-myc and anti-HER2 antibodies.

**Figure S4. UPLC-MS^E^ identification of the site of EGFR modification by 1,2-NQ.**

(*A, B*) UPLC-MS^E^ analysis results. Recombinant EGFR (5 µM) was incubated with 25 µM 1,2-NQ for 15 min at 25°C in 50 mM ammonium bicarbonate. The Glu-C-digested peptides were analyzed by UPLC-MS^E^ (see Methods). The mass data are shown in Fig. 3*A*.

**Figure S5. Modification sites in EGFR/ErbB/HER family.**

Amino acid sequence alignment in extracellular region of each EGFR/ErbB/HER family protein was shown. The red squares indicate the *N*-arylation sites in EGFR by 1,2-NQ.

**Figure S6. 1,2-NQ induces irreversible N-arylation of EGFR.**

(*A, B*) Recombinant EGFR was incubated with the indicated concentration of 1,2-NQ in the presence or absence of 1 mM DTT (*A*) or 1 mM GSH (*B*) for 30 min. *N*-Arylation by 1,2-NQ was detected with an anti-1,2-NQ antibody.
